# Supplementary figures and images for: Distance education as a tool to improve researchers’ knowledge on predatory journals in countries with limited resources: the Moroccan experience
Source: Int J Educ Integr. 2023 Jan 23;19(1):1. doi: 10.1007/s40979-023-00122-7 (PMC9868001; doi:10.1007/s40979-023-00122-7)

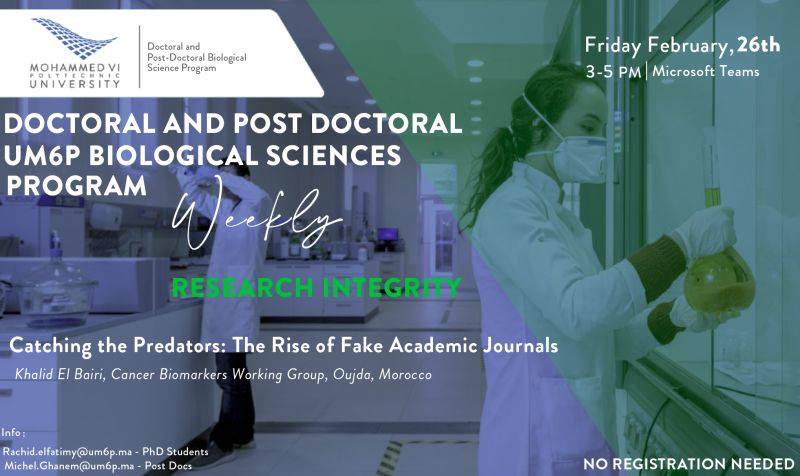


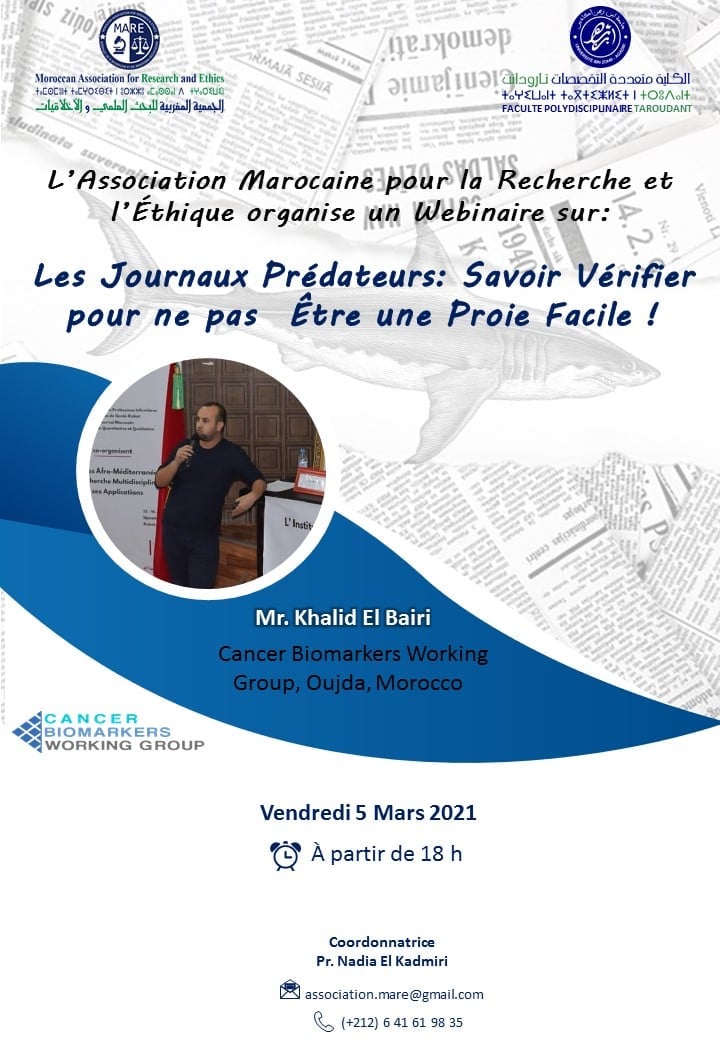

Supplement: Supplementary file 1 — Additional file 1. [file 40979_2023_122_MOESM1_ESM.docx]
