# Supplementary material for: Distance education as a tool to improve researchers’ knowledge on predatory journals in countries with limited resources: the Moroccan experience
Source: Int J Educ Integr. 2023 Jan 23;19(1):1. doi: 10.1007/s40979-023-00122-7 (PMC9868001; doi:10.1007/s40979-023-00122-7)
Supplement: Supplementary file 2 — Additional file 2. [file 40979_2023_122_MOESM2_ESM.docx]

**The Impact of Webinars in Improving Young Researchers’ Knowledge on Predatory Journals in Countries with Limited Resources: The Moroccan Experience**

L’impact des séminaires en ligne sur l’amélioration des connaissances des jeunes chercheurs sur les revues prédatrices dans les pays aux ressources limitées: l’expérience Marocaine

**2. Introduction**

**Aim:**

Exploring the impact of an online course in improving knowledge of Moroccan researchers on predatory journals and publishers

**Synopsis:**

Predatory journals are defined as fake websites claiming to be peer-reviewed academic open access journals. They publish all submitted papers in a short course of time without providing constructive peer-review. Moreover, their potential targets are authors from developing countries, encouraged by their guaranteed and rapid publication process with low open access fees. The aim of this study is to explore the impact of an advanced course regarding the predatory publishing particularly in developing countries.

Please take your time in making your decision to participate in our questionnaire. Taking part in this study is highly appreciated and voluntary. The results should be known in about 6 months from the time you are invited to take part and will be published in an international Medline-indexed journal. The questionnaire will collect basic demographic information for research purpose only. Only data from participants who will give their consents will be considered in the final analysis.

The study results will be very important to make aware of these fake journals and publication model across the world and for responsible publishing of research data.

**Conflicts of interest:**

None

**4. Data sharing and consent for publication:**

Do you give your consent to publish the collected data and communicate the final analysis in international conferences? This will be important to prevent young researchers against predatory and fake journals. By clicking forward you agree to the following:

1-All my questions have been answered

2-I agree to take part in this study

3-I give my consent to publish my data in an academic journal and make all the collected information publicly available

By responding to this survey, you will be invited to participate in free webinars with a certificate. Please do not hesitate to get in touch with us if you have any questions or ideas to improve our study (email: k.elbairi@ump.ac.ma)

**Yes**

**No**

**5. Questionnaire:**

**General information of participants**

**1-What is your gender?**

Male

Female

I prefer not to answer

**2-What is your age?**

**……..**

**3-What is your country?**

**…….**

**5-What is your degree?**

MD

PhD

MD/PhD

PharmD

PharmD/PhD

Other: please specify

**6-What is your current specialty?**

**……..**

**7-Do you consider yourself as a clinical or basic scientist?**

Clinical scientist

Basic scientist

**8-What is your current job?**

Doctor

Professor

Full time researcher

PhD student

Postdoctoral fellow

Other: please specify

**9-What is your primary institution?**

-Private sector

-Public sector

**10-Does your institution is involved in research?**

Yes

No

**11-Have you previously published any articles?**

Yes

No

**12-Do you verify the quality of a selected journal before submitting?**

Yes

No

**13-According to you, what is the journal quality parameter to be considered when submitting a research paper?**

Peer-review

Open access

Subscription-based model

The prestige of journal publishers

The journal must be published by an international oncology society

Pubmed-indexing

Scopus-Indexing

Web of Science (Clarivate Analytics)-indexing

Google Scholar-indexing

Impact factor (Journal Citation Report list)

Double indexing

Triple indexing (Pubmed, Scopus, and Web of Science)

**14-Do you feel “pressure to publish” from your supervisor, institution, and research funding agencies?**

Yes

No

I prefer not to answer

**Before participating in our webinar:**

**15-Did you know about predatory journals before this webinar?**

Yes

No

**16-Did you learn what a predatory/hijacked journal during this webinar?**

Yes

No

**17. Do you feel able to identify predatory journals after this webinar?**

Yes

No

**18. After this webinar, are you motivated to share information on predatory journals with your colleagues?**

Yes

No

**19-Have you previously been invited to publish in a predatory journal by email?**

Yes

No

**20-Have you previously published articles in a predatory journals?**

Yes

No

I don’t know if it was a predatory journal

**21-Do you think that articles published in predatory journals have the same quality as those published in peer-reviewed journals?**

Yes

No

**22-What are the reasons that encourage you to submit your research to predatory journals?**

Low open access fees

No peer-review

Fast decision

It is not important for me to be predatory or not

I don’t have time to target good journals

Recommended by my supervisor

Their invitation by emails is encouraging !

I don’t publish in predatory journals

**23-The Beall’s list was developed to help authors for not being easily scammed by predatory journals.**

**Do you check the Beall’s list of potential predatory journals and publishers before submitting?**

Yes

No

I don’t know what it is!

**24-After publishing in a predatory journal, do you feel that your efforts and research data are wasted?**

Yes

No

I prefer not to answer

I don’t publish in predatory journals

**25-According to you, why predatory journals are targeting scientists particularly from developing countries?**

I don’t know

They can afford low open access fees

They are not well informed on predatory journals

Low quality research

They like fast publication process

They are not well-informed on the importance of the peer-review process

I prefer not to answer

**26-Do you think that publishing in predatory journals has a risk on your future career?**

Yes

No

I don’t know

**27-Do you discuss the selected journal for publishing your papers with your supervisor, colleagues, co-authors, and sponsors before submitting?**

Yes

No

**28-What is your source of your publication fees of open access charges?**

Public research funds

Private sponsors

I receive fees waiver

Personal funding

I publish in subscription-based journals

**29-What do you propose to prevent young researchers against fake and predatory journals?**

-Organizing local workshops and webinars to inform researcher on this problem

-Use of social networks to inform researchers on this problem

-Research institutions should take actions against scientists that publish in predatory journals

-Surveillance of researchers by supervisors and their affiliations when submitting to academic journals should be required

**Would you like to be informed when the final data are published? If yes, please provide your email**

**Email: ………**
