# Supplementary material for: Distance education as a tool to improve researchers’ knowledge on predatory journals in countries with limited resources: the Moroccan experience
Source: Int J Educ Integr. 2023 Jan 23;19(1):1. doi: 10.1007/s40979-023-00122-7 (PMC9868001; doi:10.1007/s40979-023-00122-7)
Supplement: Supplementary file 3 — Additional file 3. [file 40979_2023_122_MOESM3_ESM.docx]

| **Features** | **% (n)** |
| --- | --- |
| **Affiliation/university**  UMP  USMBA  UIT  UIZ  UCA  UM5  UMI  UAE  USMS  UCD  UH2  UH1  UM6P  IAV  Foreign researchers^¥^  Missing data | 4.1 (9)  5.9 (13)  12.2 (27)  13.1 (29)  3.6 (8)  9 (20)  6.3 (14)  6.8 (15)  3.2 (7)  1.8 (4)  21.7 (48)  5.9 (13)  2.3 (5)  0.9 (2)  0.9 (2)  2.3 (5) |

Abbreviations: UH2: Hassan II University, UIZ: Ibn Zohr University, UIT: Ibn Tofail University, UM5: Mohammed V University, UAE: Abdelmalek Essaâdi University, UMI: Moulay Ismaïl University; UH1: Hassan I University, USMBA: Sidi Mohamed Ben Abdellah University, IAV: Hassan II Agronomic and Veterinary Institute, UM6P: Mohammed VI Polytechnic University, UCA: Cadi Ayyad University, UMP: Mohammed Premier University, USMS: Sultan Moulay Slimane University, UCD: Chouaïb Doukkali University. ^¥^Tunisia.
