# Supplementary material for: Distance education as a tool to improve researchers’ knowledge on predatory journals in countries with limited resources: the Moroccan experience
Source: Int J Educ Integr. 2023 Jan 23;19(1):1. doi: 10.1007/s40979-023-00122-7 (PMC9868001; doi:10.1007/s40979-023-00122-7)
Supplement: Supplementary file 4 — Additional file 4. [file 40979_2023_122_MOESM4_ESM.pdf]

IJEI Manuscript Report of Plagiarism

NOMBRE DE MOTS 5262

HEURE D'ENVOI 25-DÉC.-2022 11:16PM

N° DE LA COPIE 94362811

Distance Education as a Tool to Improve Researchers' Knowledge on Predatory Journals in Countries with Limited Resources: The Moroccan Experience

Abstract

The emergence of predatory journals is a global threat for scientific integrity, particularly in under-resourced settings such as low- and middle-income countries (LMICs). A bilingual course on predatory publishing using a distance education approach was developed for Moroccan researchers as a response to the imperative need for training on research ethics to implement good scientific practices. A cross-sectional survey-based study was conducted to evaluate outcomes after delivering two education sessions in both French and English. Before this course, 40% of participants indicated that they don't verify journal quality before manuscript submission and most of them consider Scopus indexing and the impact factor as the best criteria for journal publication. Approximately 50% of surveyees did not have previous information on predatory journals before the webinar. Pressure to publish by supervisors, institutions of affiliation, or funding agencies, was noticed in 43.4% of participants. After the webinar course was delivered, remarkably, the proportion of participants with knowledge on predatory publishing was meaningfully increased from 48.9% to 81%. Importantly, 83.7% of participants indicated that they are motivated to share their new knowledge on predatory publishing with their colleagues. Moreover, the majority of respondents (73.8%) reported that publishing their research in predatory journals may damage their career and professional future. Promisingly, nearly a half or more of participants recommended the use of training based on distance education, surveillance of researchers by their mentors and affiliations, and social networks to increase awareness on predatory publishing. Almost all participants were satisfied with the content of the webinar. Trainers and research institutions in LMICs should benefit from the advent of distance education using webinar platforms to increase awareness on the issue of predatory publishing in their settings.

Keywords: Webinars, Distance Education, Predatory Journals, Survey, Morocco

Introduction

During the last decade, academic publishing was considerably infiltrated by predatory journals. Their fraudulent publishing model is characterized by well-known hallmarks such as fake metrics, unsolicited mail invitations, inappropriate websites, unqualified or fabricated editorial boards, guaranteed manuscript acceptance, low article-processing charges, and particularly the nonexistence of peer-review process (Owens and Nicoll, 2019; Ruiter-Lopez et al. 2019; Cortegiani et al. 2020a; Johal et al. 2017). To date, peer-review is still the gold standard for appraising and disseminating research findings in academia, which in turn increases public's trust in science. Predatory journals bypass this process to increase acceptance rates for for-profit purposes, and they mostly accept everything submitted. Unfortunately, these so-called open access journals target authors from developing countries (Xia et al. 2014) with inaccurate training in research integrity and credible publishing practices promoted by the pressure to publish in these settings. Moreover, research findings of articles published in predatory journals are considered as not reliable and have little scientific impact (Singh Chawla, 2020). Unexpectedly, recent investigative reports have shown that predatory journals can penetrate reputed indexing databases such as Pubmed/Medline and Scopus, which can make the detection of these fake journals difficult without an adequate training (Duc et al. 2020; Severin in and Low, 2019; Cortegiani et al. 2020b, Cortegiani et al. 2020c). Furthermore, the articles of these "journals" may be displayed on Pubmed Central by individual upload of authors that use public funding under open access policies that require public deposit of sponsored research (Manca et al. 2020). Therefore, predatory journals can get into Pubmed/Medline despite that they do not meet the criteria of abstracting/indexing in this prestigious freely available database. Hence, predatory publishing is a global threat to research integrity and a serious research data wasting machine (Strong, 2019). Among various low- and middle-income countries (LMICs), Morocco appears as one of the most affected countries by publications in potentially predatory journals represented in the Scopus database (Marina and Sterligov, 2021). Providing guidance and reliable information for researchers in similar under-resourced countries seems to be a promising educational approach. Therefore, distance education holds a promise for an adequate and free-of-charge training in universities of LMICs.

During the coronavirus disease 19 (COVID-19) outbreak, distance education using webinars and e-learning platforms has remarkably gained popularity and has also emerged as a reliable solution for the disruption of face-to-face learning (Jeffries et al. 2021; Naciri et al. 2021). This innovative approach has provided promising opportunities for sharing knowledge worldwide without travel restrictions, particularly, for settings with limited resources to benefit from free advanced training. In this perspective, delivering courses on research ethics, such as the rising issue of predatory publishing, to doctoral students and researchers using distance education in LMICs may accurately remove the barriers of access to adequate research training. To date, only one paper that addressed the potential of webinars in raising awareness on predatory publishing has been published (Babb and Dingwall, 2019). This initial experience demonstrated that education sessions have the potential to increase awareness on predatory journals among health care professionals and researchers (Babb and Dingwall, 2019).

In this paper, we share our experience and outcomes of a bilingual online educational course that was delivered to Moroccan researchers to increase awareness of predatory journals and to improve their knowledge on how to critically evaluate the quality of academic publishing.

Methods

Course Development and Survey Design

Two free webinars in both English and French were developed for two different Moroccan institutions as a response to the urgent need for adequate training on how to identify predatory journals and evaluate the quality of academic publishing. The French-based education session was a certified workshop organized by the Moroccan Association for Research and Ethics/Polydisciplinary Faculty of Taroudant. This webinar entitled *“Les Journaux prédateurs: Savoir vérifier pour ne pas être une proie facile!”* was delivered on Friday 5 March 2021 using Google Meet (Google®) platform. Access to this webinar was allowed to all Moroccan researchers and students and also to some invited foreign researchers. Moreover, the webinar content was shared publicly on social networks to reach additional participation and interactions. The English-based education session was organized by the Mohammed

VI Polytechnic University (UM6P)'s doctoral and postdoctoral biological sciences program on Friday 26 February 2021. This webinar entitled *"Catching the Predators: The Rise of Fake Academic Journals"* was delivered using Microsoft Teams (Microsoft® Office 365™) as a part of the research integrity courses given to their local researchers. Course sessions were promoted using posters (**Supplemental Material 1**) and official email invitations from the organizers, reaching nearly 500 academic customers. The duration of both workshops was 2-3 hours and were all presented by the primary investigator of this paper.

The aims of the training courses were a) the general characteristics of predatory journals, b) how to identify them, c) journal indexing verification, d) salvage solutions for published science in predatory journals, and finally e) recommendations for future authors. At the beginning of the course, a historical overview of the advent of predatory journals was given as described by the famous Bohannons' landmark paper published in *Science* in 2013 (Who's afraid of Peer Review? (Bohannon, 2013)) and the Jeffery Beall's initiative on predatory publishing. This session was followed by a detailed description of predatory journals and publishers' characteristics and their infiltration of indexing databases such as Scopus, as well as the notable predatory character of an important number of Moroccan publications that were recently exposed. A third teaching unit using an online workshop on how to verify quality abstracting/indexing on Scopus, Web of Science, and Pubmed/Medline databases was completed. Moreover, the course also included an online session with cases discussion of predatory journals that infiltrate indexing databases and how to detect them as well as other particular cases such as hijacked journals. Of note, journal hijacking is based on the creation of websites and journal titles that pretend to be the real websites of legitimate academic journals (Dadkhah et al. 2016). Following this, the importance of peer-review in science and the standards of editorial workflow of academic journals was discussed. Another short session about the possible salvage solutions for already published national literature in predatory journals such as submission to Pubmed Central database was proposed to researchers in clinical and biomedical sciences. Finally, a questions and answers session ended the workshop.

A 29 questions-based cross-sectional survey (**Supplemental Material 2**) was prepared in French on Google® Forms platform. It covered 6 parts including 1)

background of the project's synopsis and objectives, 2) personal data sharing and protection and consent for publication, 3) basic demographic characteristic of participants and their general knowledge on academic publishing, 4) Pre-existing participants' knowledge on predatory journals before participating to the webinar, 5) participants' knowledge regarding predatory publishing after participating to the webinar, and finally, 6) perspectives on how predatory publishing can be avoided in LMICs and the satisfaction of the participants with the content of the workshop. Following the completion of the two workshops, the survey was distributed to course participants using their authorized registration emails. To encourage participants to fill in the form and increase the response rate to the survey, the number of questions was reduced to be limited to the most important topics that need to be addressed in LMICs. The questionnaire poll was left open for more than 6 months after the two webinars were delivered to collect voluntary responses. Reminder emails were sent one month after the survey deadline to encourage additional participation and to collect more responses. No awards were used to increase response rates. Likert scale was used to assess the satisfaction of participants with the content of the webinars.

Ethical Approval and Consent to Participate

This cross-sectional survey-based study was exempted of the requirement of ethical committee review (Moroccan Association for Research and Ethic: 15/REC/22). Electronic consents were provided by all participants before contributing to the survey. Data collection and storage were completed with respect of anonymity. The raw data of this survey are available from the corresponding author upon a reasonable request for reproducibility and transparency issues.

Data Analysis

Survey response data were downloaded on an Excel (Microsoft® Office) sheet and analyzed on IBM SPSS Statistics 25 (SPSS, Chicago, IL, USA). Data were subjected to testing for normality distribution using Shapiro-Wilk test and Q-Q plotting. We described categorical variables as percentages/absolute numbers and quantitative variables as means (±SD) and medians (± their interquartile range (IQR)) based on the results of normality testing. Multiple-choice responses were transformed into binary outcomes. Our null hypothesis (H0) was that the course delivered will not

statistically increase the proportion of participants that will identify predatory journals after webinar. The alternative hypothesis (H1) was the opposite to this previous statement. Inferential statistics using Chi-squared test or Fisher's exact test were used for bivariate analysis. We considered a two-sided P-value statistically significant when less than 0.05.

Results

General characteristics and demographic features of participants

The characteristics of the surveyed participants are summarized in **Table 1**. Nearly 500 students and researchers were invited to join these webinars. Of them, 270 participated in the educational sessions and 221 responded to the survey questionnaire with a response rate of 81.86%. Regarding the academic level of participants, a significant proportion were PhD students (68.8%; n=152), 23.1% (n=51) were PhD holders, 4.1% (n=9) were master students or with master degree, 1.8% (n=4) were MD/medical students, 1.8% (n=4) were university professors, and finally only one postdoctoral fellow participated to this course. In terms of geographic distribution, we covered all 12 public universities of the Kingdom of Morocco (n=207), a public university with private management (n=5), and also two other participants from the Hassan II Agronomic and Veterinary Institute. We also had two foreign participants from Tunisia. Missing data regarding affiliations were noticed for 5 participants. Attendees were mostly from Hassan II University (n=48; 21.7%; Casablanca), Ibn Zohr University (n=29; 13.1%; Agadir), Ibn Tofail University (n=27; 12.2%; Kenitra), Mohammed V University (n=20; 9%; Rabat), Abdelmalek Essaâdi University (n=15; 6.8%; Tetouan), Moulay Ismaïl University (n=14; 6.3%; Meknes), Hassan I University (n=13;5.9%; Settat), Sidi Mohamed Ben Abdellah University (n=13; 5.9%; Fes), followed by other institutions with low coverage (**Table 1**). Participants were 118 women (53.4%) and 103 men (46.6%) (M:F ratio 0.87) of median age of 29 years (IQR=8). Of note, despite our continuous data for age being normally distributed as demonstrated by the Shapiro-Wilk test and the Q-Q method suggesting the use of the mean, we used the median and its interquartile range because of the presence of some outliers. Of this population, 67% (n=148) of

participants did not publish any articles in academic journals before taking this course.

Knowledge of participants on academic publishing and predatory journals before taking the webinar

Responses to our survey (**Table 2**) showed that almost 40% (n=84) of participants do not verify journal quality before submission. When surveying them on the quality indicators of journals for publishing their research, Scopus indexing and Impact Factor (according to Journal Citation Reports) had the greatest importance, followed by Web of Science (WoS) indexing, peer-review, open access publishing model, Pubmed indexing, triple indexing, Google Scholar indexing, double indexing, journal publication by an international society, publisher prestige, and finally the subscription-based model having the lowest importance, respectively. Moreover, 5.4% (n=12) of participants have published their manuscripts in predatory journals before taking this webinar and 6.8% (n=15) did not know that the journals in which they published are predatory. Before participating in the webinar, approximately 50% of surveyees did not have previous information on predatory journals. When they were asked about the burden of the pressure to publish by their supervisors, their institutions of affiliation, or funding agencies, 43.4% (n=96) were affected by this issue, 31.7% (n=70) were not affected, and 24.9% (n=55) preferred not to answer this question. The survey results showed that 38% (n=84) of participants received mail solicitations from predatory journals and 9% don't know if they were predatory. Finally, 88.7% (n=196) of participants indicated that predatory journals do not have the same quality as compared to peer-reviewed journals.

Knowledge of participants on predatory publishing after the webinar

After the webinar content was delivered (**Table 3**), 81% (n=179) of participants indicated having the new basic knowledge on the characteristics of predatory/hijacked journals. Notably, the proportion of participants with knowledge on predatory publishing was statistically significantly increased from 48.9% to 81% after the webinar course was presented ($p=0.004$). In addition, 81.4% (n=180) declared that they were able to identify predatory journals based on the information provided

and the online workshop during the educational session. After the webinar, 83.7% of participants felt able and motivated to share their knowledge on predatory journals with their colleagues. When the new knowledge on predatory publishing was generated, participants indicated that the reasons for submitting research were the rapid publication process guaranteed by these journals (32.1%), the low article processing charges (24.9%), followed by the flawed/absence of peer-review (10.9%), the encouraging invitation emails (7.7%), and finally the lack of time to target good journals in addition to the supervisor's recommendation to publish in these journals (5.9% for both). The participants were also asked about the future use of the Beall's list of predatory journals before their manuscript submission, 58.4% of the 221 recorded responses indicated that they would consult this database when selecting journals. The participants have also successfully identified the most known characters of targeting scholars in LMICs by predatory journals and this includes affordable open access fees (35.7%), lack of information on predatory journals (33%), and the fact that scholars in these settings like the rapid publication process of their research (25.8%).

When examining the responses of participants regarding the risks related to publishing in predatory journals on their career, the majority of survey respondents reported that it may damage their professional future (73.8%). The remaining participants indicated not facing any career risks or they don't know (10.9% and 15.4% respectively). Importantly, nearly a half or more of respondents recommended the use of training workshops and webinars, surveillance of researchers by their mentors and affiliations, as well as social networks to increase awareness on predatory publishing. After the webinar was delivered, participants (87%) also indicated that they will not submit their research to predatory journals. However, the group of the remaining participants (13%) stated that they are under the pressure to publish even if it is a predatory journal. The satisfaction of the participants with the quality of the webinar content and their feedback toward the trainer were also surveyed at the end of the questionnaire. Overall, 49.3% were highly satisfied, 48% satisfied, 2.3% little satisfied, and only one participant was not satisfied at all.

Discussion

Legitimate publishing in academic research requires accurate education on research ethics and integrity which is one of the most important parts of the curriculum of training researchers. Early exposure of young researchers and students to this training has a central role in building good research capacities and policy to prevent serious deviations from international standards, such as publishing in predatory journals. Based on our survey, we were able, through distance education, to significantly improve the knowledge of young researchers in Morocco on predatory publishing practices. Importantly, participants were proficient in identifying most hallmarks of this issue after the webinar delivery.

The advent of novel learning technologies such as webinars during COVID-19 has massively and remarkably increased access to trainings on outstanding science that was difficult to attend before the pandemic because of geographic constraints (Bryson, 2020). More importantly, this valuable transition from face-to-face learning to online education has also enabled access to researchers from LMICs to high-quality trainings using webinars provided by research institutions and organizations from the developed world (Merritt et al. 2019). Remarkably, another notable advantage of this novel learning approach is the ability to deliver scientific contents without costs and logistic obstacles related to organizing physical face-to-face meetings and travel requirements such as visas and flights. This has promisingly increased course attendance of young scientists from LMICs to foster career development skills. Thus, distance education may enhance the transition of early- and mid-career researchers to research leaderships in these countries.

The common strategy of predatory journals to solicit submissions from researchers is accomplished via bulky spam emails (Clemons et al. 2017). In this path, our survey results revealed that ~40% of participants received mail solicitations from predatory journals and 9% did not know if they were predatory. Peer-reviewed journals rarely invite authors to submit manuscripts based on this approach, therefore, this proportion may be higher among the attendees of our webinar. Junior faculty members are particularly the target of these mails as they need publications for academic and job promotion. This category of researchers is prone to responding to these predatory journals. They expend an important amount of time to daily read and analyze these mail requests for publishing their articles, participating in predatory conferences, and also joining their editorial boards (Wilkinson et al. 2019). As

expected, Scopus indexing and Impact Factors according to Journal Citation Reports (Clarivate Analytics®) were selected by the majority of participants as the most important qualitative parameters to be considered during journal search and selection for publication. Scopus database is considered as a white list for selecting journals in many countries including Morocco. Moreover, it is also an endorsed measure for delivering funding and honoraria to researchers and it is also recommended by universities when according permissions for defending dissertations in these settings. However, Scopus contains an important amount of non-peer-reviewed literature from predatory journals (Cortegiani et al. 2020b, Cortegiani et al. 2020c), and therefore it should not be used as a unique standard when recommending good practices for publishing in academia. Unfortunately, this is the case of many institutions in LMICs such as Morocco. As such, over-reliance on the Impact Factor metric in academia has also involved some criticism (Ali, 2021; Aroeira et al. 2020; McKiernan et al. 2019). The real contribution of published science should not be based on metrics only, but also on the true impact of the findings. Thus, its potential misuse should be avoided and young researchers in LMICs trained to focus on good methods to produce actionable results.

Surprisingly, only 30.8% of the surveyed participants considered that peer-review is of importance in journal publishing. This outcome may be explained by the fact that an important number of Moroccan researchers and students are not well informed and trained about the impact of this required and decisive process in science. Indeed, they rarely benefit from workshops that provide educational sessions on epistemology in research methods and integrity. Moreover, the current doctoral programs focus more on tangible journal publications rather than the methodological rigor used by researchers to yield impactful and reproducible findings. In fact, this is in line with the widely practiced “publish” or “perish” concept (Barrett, 1962; Guraya et al. 2016; Al-Adawi et al. 2016). This movement has caused more negative collateral consequences to LMICs such as predatory publishing and the remarkable attention given to bibliometric indicators, as well as the focus on “publishability” rather than the value of the content of local research. This also raises concerns about the misuse and distortion of the journal Impact Factor and Indexing in academic publishing. Recently, several Moroccan universities have launched an honorarium program to reward researchers and their affiliations based on bibliometric indicators

of their publications (https://leseco.ma/sciences/luniversite-sidi-mohamed-ben-abdellah-lance-une-prime-a-la-production-scientifique.html). According to some Moroccan researchers, this grant package may positively affect their research and satisfaction of the research environments in local universities. However, rewarding researchers based on these metrics only rather than on the impact of their findings and quality may negatively predict a future in which publications are more important than research findings and methods quality. This harmful pay-per-publication system based on allowance programs is well known in other countries (Quan et al. 2017; McKiernan et al. 2019) and has just arrived in some LMICs such as Morocco. Therefore, the creation of academic funding agencies rather than these aberrant monetary reward systems is awaited to receiving funding to merited projects and build academic skills that may improve country development indicators.

In addition to the previous observations, targeting journals that use subscription-based model had the lowest importance to participants in this survey despite that it is the most adapted publishing model for LMICs. This may be due to the emergence of "open access" which is nowadays increasingly being used worldwide including LMICs as compared to the traditional subscription-based scholarly publishing (Matheka et al. 2014; Singh et al. 2021). Open access publishing requires funding which, in the case of respectable open access journals, involve high publishing fees that are not affordable by authors in LMICs (Singh et al. 2021; Newton, 2020). Thus, cheap open access as supported by predatory publishing in LMICs is the most adopted solution in these settings as widely reported. Hence, there is an urgent need to train scientists in LMICs on this issue and also reframe the whole process of open access publishing to address this ongoing problem. Stringent and reasonable criteria should be developed to unravel the real cost of publishing in open access journal; which, in most cases, is overestimated and not well justified by publishers. The absence or the low quality of peer-review in predatory journals is only a piece of the puzzle of this issue. The so-called open access indexed and peer-reviewed journals with established academic publishers may also have predatory behaviors as well; including racism (Niriella et al. 2021), flawed or decreased quality peer-review process (Van Vlokhoven, 2019; Erfanmanesh, 2017; de Jong, 2017), and remarkably the high fees of open access proposed to researchers in emerging economies. This

raises the question of categorizing peer-reviewed journals that place constraints to science from LMICs into predatory journals.

Although incomplete and questionable for some cases of potentially predatory journals, the Beall's list is still widely used by academicians and students to categorize fraudulent publishers and predatory journals (Strielkowski, 2018; Strielkowski, 2017). The currently updated list of predatory journals (https://beallslist.net/) is consulted for research purposes and also by authors wishing to verify journals. This trend was also noticed for our surveyed participants with more than a half of them indicating that they will routinely use this list to identify predatory journals and publishers.

The phenomenon of "predatory scholars" is another issue in LMICs. Researchers who have developed their curriculum based on predatory publishing may become university professors and also decision makers. Once becoming principal investigators and mentors in academia, the likelihood of publishing in predatory journals by their students tends to be higher, which in turn promotes the spread of this attitude in junior researchers. In our survey, 13 participants (5.9%) indicated that they published in predatory journals because it was recommended by their supervisor which might also be in line with this observation. Publishing in predatory journals may harm the professional career of researchers and their reputation (Ward, 2016; Shrestha, 2020). A recent study investigating the perception by PhD students of predatory journals found that such research ethics violation had a negative impact on their career (Wang et al. 2021). This outcome is similar to our findings and other authors' results (Rawas et al. 2020), suggesting that predatory publishing can damage career development.

Remarkably, the results of our survey showed that most participants were motivated to share their knowledge on predatory journals with their colleagues. Moreover, they acknowledged that they were committed to avoid submitting their research to predatory journals after the webinar delivery; suggesting that this distance education approach was effective in increasing awareness on the harms that predatory publishing may cause. The rest of participants (13%) indicated that pressure to publish is still notable and they will submit their manuscripts to these journals. In fact, this behavior from scientists feeling high pressure to publish was widely reported as a

principal factor affecting “publishability” in predatory journals (Rawas, et al. 2020; Cobey et al. 2019). A recent systematic review of the causal factors that affect the decision of authors to publish in predatory journals confirmed that publication pressure contributes to this phenomenon (Mertkan et al. 2021). This tendency was noticed in research environments in which authors have a limited ability to publish in reputed journals (Mertkan et al. 2021). Similarly, our survey results showed that Moroccan researchers are also affected by this problem of pressure to publish by their mentors, affiliations, or funding agencies suggesting that this is not limited to developed countries.

Our study has a number of strengths and also some limitations. To the best of our knowledge, only one tutoring session based on distance education for increasing awareness on predatory publishing is available for citation (Babb and Dingwall, 2019). This was a webinar that was developed for Canadian health professionals (n=33) to improve their knowledge on predatory journals and how to critically review the medical literature. Our education sessions were developed with two languages and were of the highest enrollment to date (n=221). We covered all Moroccan public universities which is also promising for knowledge sharing between researchers. Notably, awareness on predatory publishing was considerably improved after our webinars. Most importantly, the majority of participants were PhD students and had not published any papers to the date of the online workshop which is promising to prevent any infiltration of predatory journals to their future career. Moreover, these attendees were motivated to share their novel knowledge with colleagues that did not participate in this educational session. This is of high importance to continue the discussion in national research institutions for a wide reporting of this issue. However, despite these encouraging outcomes, the proportion of participants seems to be low when compared with the number of registered PhD students in Moroccan universities. Remarkably, we failed to attract university professors to this webinar (4 only). This category of decision makers has the potential to effectively impact the research work environments as compared to junior researchers. We also failed to have MD/MD students in our educational course (n=4), especially that they belong to the most affected fields in terms of publishing in predatory journals (Marina and Sterligov, 2021). Remarkably, while most participants indicated that they would use the newly learnt information in their future publishing behavior, it is difficult to

evaluate whether they actually did or not. Conducting a longitudinal study would have been more effective to inquire about how gained knowledge from this educational intervention will be used. Finally, another limitation is the design and the single-country nature of our cross-sectional survey-based study which may not represent a global viewpoint of LMICs.

Some perspectives and recommendations can be shared after these preliminary outcomes of distance education to join the global fight against the threat of predatory publishing. Training on predatory publishing, research integrity and how to critically appraise academic literature must be institutionalized. Real engagement of youth as trainees and also as trainers is needed to fight against scientific malpractice in LMICs. Open science through open access -this false gold- requires institutional funding. Indeed, LMICs cannot produce practice-changing findings with the use of current open access journals that in most cases are predatory and in other cases put barriers to authors from these settings. Therefore, one of the truthful solutions is to halt the transformation of hybrid journals into fully open access and allow authors from LMICs to share their accepted non-edited publications without legal copyright restrictions. Building associations and organizations, such as *Moroccan Association of Research and Ethics* that organized one of these courses, may have a remarkable role in training young scientists in LMICs by providing free webinars and workshops to implement good education on research methods and ethics. Moreover, creating research integrity committees to survey researchers' practices in LMICs and centers of "journalology" to deliver recommendations on where local science should be published are awaited. The Canadian Center of Journalology of The Ottawa Hospital (http://www.ohri.ca/journalology/predatory-journals) is a good example of these initiatives and frameworks that should also be implemented in under-resourced countries as it requires well-trained teams only.

Another issue that promotes the proliferation of predatory publishing in LMICs is the low quality of research conducted in these resource-constrained environments. This is the principal reason for not attracting good academic journals of the developed world to consider LMICs-based science for publication. LMICs may benefit from the current advances in scholarly publishing transparency such as the emergence of the open peer-review initiative which will certainly offer an approach to improve the quality of local science. Unpublishable science from LMICs that is lost in predatory

journals should be published in journals of LMICs developed with high standards of methodological and ethical rigor instead of waiting for highly impacted journals to take care of their settings-associated problems. This is achievable in the digital era; conditioned by accurate training of the marginalized youth in LMICs. In this perspective, investing in people to drive research and train the research workforce using the advantages of distance education may help in reaching this goal. Absolutely, we all rely on publications to improve countries development indicators such as health, but rigor in generating and publishing data is required.

Conclusion

Students and early-career researchers have limited exposure to training on research integrity which is lacking or inadequate in LMICs. This makes local researchers a hot target for the attack of trash science publishers. However, there is a shifting transition in terms of delivering efficient training research workshops to early-career investigators in LMICs towards a global education goal. According to the findings of our study, the impact of webinars in the learning process of young researchers is encouraging. Distance education has the potential to offer free training to scientists for increasing awareness on predatory publishing in LMICs. Moreover, preparing courses on research ethics, best publication practices, and research methodology is inexpensive to implement in low-resourced countries. Similar initiatives and programs from other LMICs are awaited to explore the issue of predatory journals widely and to build human resource capacities.

IJEI Manuscript Report of Plagiarism

RAPPORT D'ORIGINALITÉ

2 %

INDICE DE SIMILARITÉ

SOURCES PRINCIPALES

1	Khalid El Bairi, Nadia El Kadmiri, Maryam Fourtassi. "Exploring Scientific Misconduct in Morocco Based on an Analysis of Plagiarism Perception in a Cohort of 1,220 Researchers and Students", Accountability in Research, 2022<br>Crossref	59 mots — 1 %
2	bmccancer.biomedcentral.com<br>Internet	14 mots — < 1 %
3	www.dovepress.com<br>Internet	12 mots — < 1 %
4	Qiutong Yu, Yuqing Cheng, Wei Li, Genyong Zuo. "Mediating Factors Explaining the Associations between Solid Fuel Use and Self-Rated Health among Chinese Adults 65 Years and Older: A Structural Equation Modeling Approach", International Journal of Environmental Research and Public Health, 2022<br>Crossref	10 mots — < 1 %
5	www.frontiersin.org<br>Internet	10 mots — < 1 %
6	Kassahun Haile, Solomon Kebede, Temesgen Abera, Abebe Timerga, Ayenew Mose. "Thrombocytopenia Among Pregnant Women in Southwest Ethiopia: Burden, Severity, and Predictors", Journal of Blood Medicine, 2022	9 mots — < 1 %

Crossref

EXCLURE LES CITATIONS	ARRÊT	EXCLURE DES SOURCES	ARRÊT
EXCLURE LA BIBLIOGRAPHIE	ARRÊT	EXCLURE LES CORRESPONDANCES	ARRÊT
